# Exploratory Analysis of Objective Outcome Measures for the Clinical Assessment of Erosive Tooth Wear

**DOI:** 10.3390/diagnostics13152568

**Published:** 2023-08-02

**Authors:** Maria Jacinta Rosario H. Romero, Peter S. Ungar, Daniel Fried, Frank Lippert, Domenick T. Zero, Susan Zunt, George J. Eckert, Ana Gutierrez Gossweiler, Dylan Jacob Elkington-Stauss, Guillermo Tamayo-Cabeza, Adam B. Kelly, Troy Bartels, Camille Kita, Elizabeth Wewers, Anderson T. Hara

**Affiliations:** 1 Department of Cariology, Operative Dentistry and Dental Public Health, Indiana University School of Dentistry, Indianapolis, IN 46202, USA; mhromero@iu.edu (M.J.R.H.R.); gtamayo@iu.edu (G.T.-C.); 2 Restorative Dentistry Section, Department of Clinical Dental Health Sciences, College of Dentistry, University of the Philippines Manila, Manila 1007, Philippines; 3 Department of Anthropology, University of Arkansas, Fayetteville, AR 72701, USA; pungar@uark.edu (P.S.U.); djelking@uark.edu (D.J.E.-S.); tdbartel@uark.edu (T.B.); cekita@uark.edu (C.K.); ewewers@uark.edu (E.W.); 4 Department of Preventive and Restorative Dental Sciences, University of California San Francisco, San Francisco, CA 94143, USA; daniel.fried@ucsf.edu; 5 Oral Health Research Institute, Department of Cariology, Operative Dentistry and Dental Public Health, Indiana University School of Dentistry, Indianapolis, IN 46202, USA; flippert@iu.edu (F.L.); dzero@iu.edu (D.T.Z.); amgutier@iu.edu (A.G.G.); 6 Department of Oral Pathology, Medicine and Radiology, Indiana University School of Dentistry, Indianapolis, IN 46202, USA; szunt@iu.edu; 7 Department of Biostatistics, Indiana University School of Medicine, Indianapolis, IN 46202, USA; geckert@iu.edu; 8 Oral Health Research Institute, Indiana University School of Dentistry, Indianapolis, IN 46202, USA; abkelly@iu.edu

**Keywords:** erosive tooth wear, dental enamel, optical coherence tomography, enamel surface texture, BEWE

## Abstract

This study proposed using enamel surface texture and thickness for the objective detection and monitoring of erosive tooth wear (ETW), comparing them to the standard subjective Basic Erosive Wear Evaluation (BEWE). Thirty-two subjects (*n* = 597 teeth) were enrolled in this longitudinal observational clinical study. Enamel thickness (by cross-polarization optical coherence tomography, CP-OCT) and 3D dental microwear parameters, i.e., area-scale fractal complexity (Asfc), anisotropy (Str), and roughness (Sa) (by white-light scanning confocal profilometry), were obtained from buccal surfaces. Buccal, occlusal, and lingual surfaces were scored for BEWE and the maximum score per tooth (BEWE_Max_) was determined at baseline and 12 months (M12). Data outcome relationships were evaluated (alpha = 0.05). Enamel thickness decreased (*p* < 0.001), BEWE scores, Sa, and Str increased (*p* < 0.001), while Asfc did not change at M12. Baseline BEWE_Buccal_ correlated strongly with BEWE_Max_ (r = 0.86, *p* < 0.001) and moderately with BEWE_Lingual_ (r = 0.42, *p* < 0.001), but not with enamel thickness (r = 0.03, *p* = 0.43). Change (Δ) in surface texture outcomes correlated poorly but significantly with ΔBEWE_Buccal_ (r = −0.15–0.16, *p* < 0.001) and did not correlate with Δenamel thickness (r = 0.02–0.09, *p* > 0.06). Teeth with BEWE progression revealed a greater increase in ΔSa and ΔStr. These findings suggest that enamel surface roughness can potentially determine ETW severity, and CP-OCT may be relevant for clinically monitoring enamel thickness.

## 1. Introduction

Erosive tooth wear (ETW) is a dental condition that results in loss of tooth structure as a consequence of chemo-mechanical wear processes [1]. Acids that are present in the oral cavity from intrinsic (gastric) and/or extrinsic (dietary) sources [2] can soften exposed enamel and dentin surfaces and make them vulnerable to wear from the abrasive forces of mastication and toothbrushing. Given the irreversible nature of ETW, tooth form, esthetics, and function can be compromised [3]. ETW has a high prevalence worldwide [4] and it affects approximately 46% of teenagers [5] and 80% of adults [6] in the United States, as reported by the National Health and Nutrition Examination Survey (NHANES). Despite these alarming numbers, specific diagnostic rules and evidence-based management guidelines are yet to be established. Currently, clinical assessment and monitoring of ETW are performed by visual examination using subjective indices [7]. This traditional approach limits the detection of ETW lesions to mostly advanced stages, wherein considerable destruction of the tooth has already occurred, resulting in pain and irreversible changes in dental form, function, and esthetics. In these circumstances, the required restorative treatments are complex and costly [8]. There is a clear need for objective methods to detect, diagnose, and monitor ETW early, allowing the implementation of personalized and evidence-based management plans focused specifically on preventive measures. 

This exploratory analysis involved subjects previously diagnosed with hyposalivation. Saliva plays a crucial role in the development of ETW, and the lack thereof puts patients at higher risk for ETW development or progression [9,10]. Our first hypothesis in this investigation was that ETW lesions could be detected and differentiated by using scale-sensitive, tridimensional dental surface texture analysis of point clouds generated by white-light scanning confocal profilometry (WSCP). Some integrated metrics of dental microwear analysis have proven to be useful for similar dental applications, including surface fractal complexity (or change in apparent roughness with the scale of observation) and anisotropy (or directionality of the wear pattern) [11]. Our previous in vitro and in situ data have shown the ability of these outcomes to identify and differentiate between the main dental wear mechanisms of ETW lesions with a good degree of certainty [12,13]. Our second hypothesis was that cross-polarization optical coherence tomography (CP-OCT) could provide objective clinical measures of ETW progression, based on the longitudinal monitoring of dental enamel thickness. CP-OCT allows for safe, non-destructive, and repetitive measurements of enamel thickness [14]. It also allows for the study of the tissues’ polarization properties and provides higher-resolution images and better visualization of the enamel structure and dentin–enamel junction [15,16]. Our previous in vitro and in situ studies have demonstrated its potential as a clinical tool to monitor ETW [17,18]. 

This exploratory study aimed to compare the objective outcomes of enamel surface texture using WSCP and enamel thickness with CP-OCT to a standard subjective visual assessment (Basic Erosive Wear Evaluation—BEWE) and explore their potential to be used clinically for the detection and monitoring of naturally developed ETW lesions in a high-risk population. 

## 2. Materials and Methods

### 2.1. Study Design

This study consisted of a single-site longitudinal observational clinical study, conducted at the Oral Health Research Institute of the Indiana University School of Dentistry (IUSD). Twenty-nine subjects previously diagnosed with dry mouth at The Center for Oral Diagnosis and Treatment, IUSD, or any other IUSD clinic, and three control (no dry mouth) subjects were enrolled to participate in the study. The study was carried out for about 20 months employing a ‘rolling’ subject recruitment that lasted for six months. Each recruited subject was then assessed periodically up to 12 months after their baseline visit. 

A trained and calibrated examiner performed the clinical assessment using the BEWE index to classify the surfaces of all incisors, canines, premolars, and first molars according to the degree of severity of the ETW lesion (score 0: no erosion; 1: loss of enamel surface texture; 2: enamel loss of <50% of surface area; 3: enamel loss of >50%) and a self-completed questionnaire (diet and behavioral habits) was used to identify related risk factors. Subjects at high risk for ETW development were selected for the study based on these criteria. Further evaluation of the subjects’ teeth by trained examiners was performed using the test objective outcomes of surface texture and enamel thickness. Outcomes were assessed at baseline and after 3 (M3), 6 (M6), 9 (M9), and 12 (M12) months.

### 2.2. Study Participants

The clinical study protocol followed the Declaration of Helsinki and was reviewed and approved by the Indiana University-Purdue University Indianapolis Institutional Review Board (protocol no. 1910664803). Subject recruitment and screening occurred between July 2020 and February 2021. Potential subjects previously diagnosed with dry mouth at The Center for Oral Diagnosis and Treatment (CODT, IUSD) were invited to participate in the study. The control subjects (no diagnosis of hyposalivation) were recruited from the IUSD dental clinics. These subjects were prescreened to meet age and health requirements using an IRB-approved phone script.

Prior to screening, subjects were asked to sign a written informed consent form. To be included in the study, subjects had to be 18–85 years old and generally healthy; have a minimum of eight BEWE-scorable teeth with at least one ETW lesion (BEWE ≥ 1), except for control subjects; have indicated dietary acid exposure in the questionnaire; and have been previously diagnosed with dry mouth, except for controls. Control subjects needed to have a normal salivary flow rate of ≥0.8 mL/min stimulated and ≥0.2 mL/min unstimulated saliva and teeth without clinical signs of advanced ETW. Subjects were excluded if they were visually assessed by the study dentist to clinically have any untreated cavitated caries lesions or moderate to severe periodontal disease. Subjects who presented with caries lesions at screening were only allowed to continue in the study if they had their caries lesions appropriately treated before the baseline visit. 

The sample size calculation indicated the need to recruit 60 subjects with a higher risk for ETW (dry mouth) and 8 controls (normal salivary flow). We assumed 8 teeth per subject, with baseline BEWE scores in high-risk subjects as follows: 10% of teeth with BEWE 3, 20% with BEWE 2, 30% with BEWE 1, and 50% with BEWE 0. The 95% lower confidence bound (LCB) for overall model accuracy to classify teeth by baseline BEWE score extends 3% from the estimated accuracy; within each BEWE score, the LCB extends up to 10%. The expectation was that at least 10% of teeth would show ETW progression, so the LCB for accuracy of the model predicting ETW progression extends up to 10% from the estimated accuracy.

### 2.3. Clinical Study Procedures

Study completion entailed subject participation during each of the six study visits: screening, baseline, M3, M6, M9, and M12. Study participants were asked for informed consent and their medical history as well as the completion of a self-administered diet and behavioral questionnaire. Oral soft and hard tissue examinations were conducted and clinical assessment using the BEWE index was performed by a trained/calibrated examiner to determine which subjects qualified for the study. Qualified subjects had alginate impressions (Jeltrate Plus, Dentsply Caulk, Milford, DE, USA) taken for both dental arches that were used for the fabrication of CP-OCT tray guides (described below) and modified custom trays. Unstimulated and stimulated saliva flow rates were measured during the initial visit for the control group subjects. Qualified subjects returned after 1 week (±5 days) for the continuation of the baseline assessments. Salivary flow rates were then determined for the dry-mouth subjects during the subsequent visit after discontinuing their saliva-stimulating medications 24 h prior. The subjects’ teeth were brushed using a prepasted toothbrush (ReadyBrush, ReadyBrush, Boca Raton, FL, USA), rinsed, and dried to remove the biofilm or pellicle. Dental impressions were taken for both dental arches of all the subjects using polyvinylsiloxane impression material (President Jet Regular and Light Body, Coltene/Whaledent AG, Altstätten, Switzerland) for enamel surface texture analysis. Three-dimensional CP-OCT images were then taken from the window of the CP-OCT imaging tray guide for the measurements. Each of the assessment methods for ETW (BEWE, diet and behavioral questionnaire, CP-OCT enamel thickness, and surface texture) was conducted during the succeeding visits at M3, M6, M9, and M12. 

### 2.4. BEWE Scoring

ETW severity was scored and recorded according to the BEWE index [19]. A previously trained examiner scored the buccal, occlusal (for posterior teeth), and lingual surfaces of all incisors, canines, premolars, and first molars according to the degree of severity of the ETW lesion. Scoring was performed at baseline, M3, M6, M9, and M12.

### 2.5. Surface Texture Analysis

White-light scanning confocal profilometry (Neox, Sensofar LLC, Newington, CT, USA) was used to analyze the buccal surface of the impressions. The central area of the middle third (mesiodistally and inciso/occluso-cervically) of the buccal surface was chosen for analysis. The planimetric work envelope for each tooth sample was 242 × 181 µm^2^ with a lateral point spacing of 0.17 μm in both x and y directions, a vertical step of 0.2 µm, and a published resolution of <2 nm, measured in three locations, as previously reported [12]. Trained and calibrated examiners performed measurements on the central area of the buccal surfaces of specimens collected at baseline and M12. Point clouds for each surface were assessed by dental microwear texture analysis. Area-scale fractal complexity (Asfc) [20] and ISO 25178 standards for texture aspect ratio (Str) and arithmetical mean height (Sa) were calculated to characterize scale-sensitive complexity, anisotropy, and roughness of each surface, respectively. Analyses were conducted using MountainsMap 8 (Digital Surf, Besançon, France). These variables have been shown to consistently reveal aspects of the surface texture of value for distinguishing dental wear types [12,21,22].

### 2.6. Enamel Thickness Analysis

**Figure 1:**
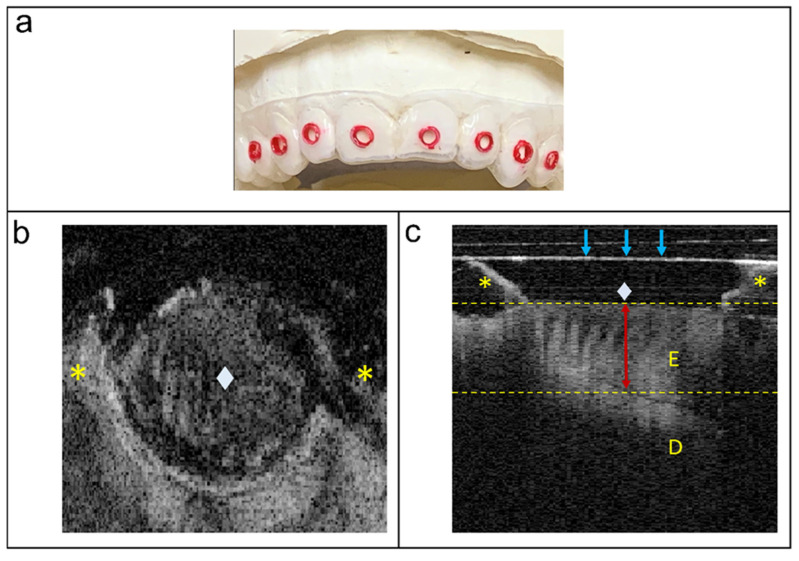
CP-OCT imaging of the buccal surface of a maxillary molar. (**a**) Anterior view of a maxillary CP-OCT imaging tray guide. (**b**) Top view. (**c**) B-scan. E: enamel; D: dentin. * CP-OCT tray guide; ♦ enamel surface within the 2 mm round CP-OCT tray guide window. Blue arrows: CP-OCT probe window with a plastic barrier; red arrow: area measured for enamel thickness which is the midline of the enamel width as measured from the base of the CP-OCT tray guide window.

CP-OCT imaging tray guides (Figure 1a) were custom-made to fit the subjects’ maxillary and mandibular dental arches using soft ethylene vinyl acetate sheets (125 mm round, 1 mm thick, Keystone Industries, Gibbstown, NJ, USA). The sheets were vacuum-formed to the casts of each dental arch using a pressure molding machine (Biostar^®^ SCHEU-DENTAL GmbH, Iserlohn, Germany) and trimmed to extend half a tooth away from the most posterior tooth included in the study and about 2 mm away from the gingival margin to maintain good adaptation. Round window holes 2 mm in diameter were created on the middle third (mesiodistally and inciso/occluso-cervically) of the buccal surfaces. A thin layer of red nail varnish was applied onto the walls of the CP-OCT tray guide windows for easier localization while positioned in the subject’s oral cavity. The same CP-OCT imaging tray guides with round windows were used for each subject over the different study visits (while observing infection control measures) to facilitate CP-OCT probe repositioning and imaging of the same area on the buccal surfaces of the tooth over time to be used for longitudinal enamel thickness analysis. 

Three-dimensional enamel scans were acquired using a portable dental CP-OCT system with a handheld probe (Santec Inner Vision IVS-300-S-L-C; Santec Corp., Komaki, Japan). The device used a swept source laser light with a center wavelength of 1310 ± 30 nm and a high scan rate of 30 kHz with a maximum lateral probe scanning area of 5 × 5 mm and a working distance of 1 mm. Axial imaging in the air was >5.6 mm with a 3 mm depth of focus while axial and lateral resolutions in the air were ≤12 µm and 30 µm, respectively. 

Before scanning, the buccal surfaces of the maxillary teeth were gently air-dried for ~10 s and isolated using cotton rolls as needed. The CP-OCT imaging tray guide was then fitted on top of the teeth and 3D CP-OCT scanning was carried out. During scanning, the probe was held horizontally perpendicular to the long axis of the tooth being scanned and positioned directly on top of the CP-OCT tray guide with the borders of the CP-OCT tray guide window contained within the scanning area. Three-dimensional tomograms were obtained from the buccal surfaces of each of the included maxillary teeth using a dedicated imaging and analysis software (Inner Vision IVS-300 ver5.3.2, Santec Corp., Komaki, Japan) where the refractive index was set at 1.6 for enamel. Once the maxillary arch was scanned, the maxillary CP-OCT imaging tray guide was removed, and the same methods were applied to the mandibular arch. Central B-scans (2D images) in the Y-direction were then selected from each 3D scan and saved for enamel thickness analyses. Enamel thickness measurements (from DEJ to the surface of the specimen) on the 2D images were performed using Santec Inner Vision IVS-300 software (Santec Corp, Komaki, Japan). The measurement position was identified at the center of the enamel width at the base of the CP-OCT tray guide window with the aid of a screen ruler (A Ruler for Windows v3.3, https://www.arulerforwindows.com accessed on 8 July 2022). The distance (mm) between the depths of the highest light intensity peaks at the enamel surface and DEJ areas was then calculated from the A-scan. CP-OCT scanning and enamel thickness measurements were performed at baseline, M3, M6, M9, and M12. A representative B-scan of a maxillary molar is shown in Figure 1c.

### 2.7. Statistical Analyses

Data collected at baseline and M12 for all parameters were used for the analyses. Data for M3, M6, and M9 were excluded as surface texture parameters of Asfc (complexity), Str (anisotropy), and Sa (roughness) could not be obtained.

Spearman correlation coefficients were used to evaluate the associations among measurements. Using the longitudinal follow-up assessments, linear mixed-effects models evaluated changes in surface texture and enamel thickness (monitor ETW). These models included random effects to correlate data within a tooth over time, as well as allow different variances at each time point and correlate multiple teeth within a subject. Teeth with changes in BEWE scores were compared to those without such changes during follow-up for differences in changes in surface texture and enamel thickness parameters using linear mixed-effects models. A two-sided 5% significance level was used for all tests.

## 3. Results

Twenty-nine subjects with a history of diagnosed hyposalivation and three control subjects were enrolled in the study. One dry-mouth subject dropped out after the baseline visit. A maximum number of 597 teeth that were scoreable for BEWE on any of the surfaces at baseline were included. Among them, 584 teeth were used for surface texture analyses of their buccal surfaces, and 531 teeth were included for the enamel thickness measurements with CP-OCT. Teeth that were malposed or posteriorly located with the buccal mucosa not allowing proper CP-OCT probe positioning were excluded. Out of the 29 subjects who had a history of hyposalivation, 9 and 18 had normal unstimulated and stimulated saliva flow rates, respectively. 

BEWE scores of the different surfaces (BEWE_Buccal_, BEWE_Occlusal_, BEWE_Lingual_) at baseline and M12 of dry-mouth subjects are shown in Table 1. BEWE for all surfaces was greater at M12 compared to baseline (*p* < 0.001). For the same longitudinal comparison, enamel thickness decreased, Asfc showed no change, and Sa and Str parameters increased. Results for the different parameters for control subjects are shown in Table S1. Statistical analyses within the control group, as well as the comparison of the control and dry-mouth subjects, were not performed due to the small number of control subjects recruited.

The direct comparison between tested parameters (Table 2) revealed that BEWE_Occlusal_ correlated poorly with enamel thickness as measured on the buccal surface of posterior teeth. BEWE_Buccal_, on the other hand, did not correlate with enamel thickness measurements. BEWE_Buccal_ and BEWE_Lingual_ correlated moderately, while BEWE_Buccal_ correlated strongly with the BEWE_Max_. Comparisons of the longitudinal changes indicated that ΔAsfc and ΔStr correlated moderately, while ΔAsfc and ΔSa, as well as ΔSa and ΔStr, exhibited weak correlation (Table 3). Both ΔBEWE_Buccal_ and ΔBEWE_Lingual_ correlated moderately with ΔBEWE_Max_. A similar correlation was observed between ΔBEWE_Occlusal_ (posterior teeth) and ΔBEWE_Max_. Only data at baseline and M12 were compared and analyzed. 

Additional analyses comparing teeth with and without ETW progression—based on BEWE examination—revealed no significant differences in ΔAsfc and Δenamel thickness over time. However, teeth with ETW progression (BEWE increase) showed greater changes in ΔSa and ΔStr over time compared to teeth without ETW progression (Table 4). 

## 4. Discussion

Our previous in vitro [12,18] and in situ studies [13,17] have demonstrated the potential of enamel thickness (by CP-OCT) and the dental microwear texture parameters Asfc, Str, and Sa (by WSCP) to detect and monitor ETW lesion progression over time. However, in vitro and in situ studies are limited as they do not fully replicate clinical conditions. In the present exploratory clinical study, these objective outcomes were compared to the BEWE index, a subjective but widely used clinical index for ETW. The study population consisted of patients previously diagnosed with hyposalivation, as they were considered at higher risk for ETW, and their clinical findings were to be compared to those of subjects who were never diagnosed nor had symptoms of hyposalivation (control). Unfortunately, our planned recruitment and part of the laboratory analyses (dental microwear) were negatively impacted by the restrictions imposed by the COVID-19 pandemic. Even after extending the recruitment phase from 3 to 6 months, it was not feasible to meet our total target subject numbers (*n* = 68). A decision was made to stop enrollment, considering that we had reached the number of teeth needed, as determined by our a priori sample size calculation (*n* = 544). This was possible as we had initially set the minimum number of scoreable teeth per subject at eight, but most subjects had a significantly higher number of teeth that were scoreable with BEWE. However, it is important to note that the lower number of enrolled subjects may have limited the individual subject variation representation in our data. Nevertheless, the varied nature of ETW per tooth surface within the same subject supported the shift to using the total number of teeth instead of the number of subjects as the basis for the sample size. In addition to the aforementioned limitation brought about by the smaller number of subjects, the initially planned comparison between hyposalivation and control participants (at *n* = 3), wherein ETW progression was less expected, was no longer applicable and is thus a limitation of this study. Tooth-based comparisons between teeth with and without ETW progression within the hyposalivation group were performed instead to see the trends and associations among the different ETW parameters (Table 4). 

In the current study, all surfaces of each tooth included were scored for BEWE, and the BEWE_Buccal_ scores were compared to those of the other parameters. Progression of ETW over time was observed in the hyposalivation population, as shown by the significant increase in BEWE scores from baseline to M12 for all surfaces (*p* < 0.001). Nonetheless, the mean increase in the BEWE score was less than 1 and smaller than what we anticipated for high-risk subjects. In hindsight, a longer study duration could have been advantageous to better discern between subjects at high and low risk for ETW. Although recruited subjects had been previously diagnosed with hyposalivation, and were therefore at higher risk for ETW [9,10], 9 showed normal unstimulated salivary flow, while 18 out of the 29 subjects had normal stimulated flow rates during testing, despite temporarily discontinuing their saliva-stimulating medications at least 24 h prior to the measurements. This might indicate that for these subjects, dry mouth may not have been a major risk factor for ETW. Further differentiation of the subjects based on their salivary flow rates was not performed since the related inclusion criterion was only a history of hyposalivation. Moreover, salivary flow rates could have varied during the length of the study due to the adoption of individual measures to control the clinical symptoms or changes in the severity of the condition [23]. Future studies ensuring a larger number of subjects and periodical measurement of actual salivary flow rates may provide better insights into the differences in effects of an earlier hyposalivation diagnosis and specific salivary flow rates on ETW progression. In terms of surface comparison, change in BEWE was greater for the occlusal and lingual surfaces than for the buccal surface, which means that ETW progression was more evident on both occlusal and lingual surfaces than the buccal. However, BEWE on all three surfaces correlated moderately with BEWE_Max_ (Table 2). This supports the use of buccal surfaces for comparison with the other test parameters in this study. 

The presence of ETW progression was also supported by the decrease in enamel thickness from baseline to M12 as measured by CP-OCT. This corroborates CP-OCT’s potential to monitor changes in enamel thickness both in vitro and in situ within the same tooth over time [17,18]. These prior studies [17,18] were carried out in ideal conditions, with enamel specimens of standardized dimensions and flat enamel surfaces allowing proper working distance and probe angulation during imaging. Despite the encouraging results observed in the current study, there were many challenges. Our measurements were limited to the buccal surfaces only as the wide and flat probe head configuration made the access of lingual surfaces challenging. Also, clinical CP-OCT imaging was limited by different surrounding anatomical structures, such as the oral soft tissues. As mentioned earlier, teeth that are malposed or with oral soft tissues precluding proper CP-OCT probe positioning were excluded, resulting in relatively fewer teeth examined for the enamel thickness parameter in the study. The natural curvature of teeth as well as the involvement of the whole tooth contributed to greater difficulty in probe positioning. Hence, the use of a CP-OCT tray guide with pre-established windows was essential in ensuring more accurate repositioning and imaging of the same area of each tooth during every visit. The scanning window of the CP-OCT probe was held as close as possible and parallel to the buccal surface of the CP-OCT tray guide during scanning. Nevertheless, it was inevitable to have some degree of variation during imaging. In such cases, there were slight differences in the angulation of the surface being scanned. This also could have had an effect on the variability and the ease of obtaining enamel thickness measurements at the center of the enamel width captured within the CP-OCT tray window from the enamel surface to the DEJ. For teeth in which some angulation variation was unavoidable, particularly in the molars and malposed teeth, uneven light attenuation was more evident with the unequal working distance from the enamel surface. A greater distance meant higher attenuation and a lower contrast of enamel from the underlying structures, which made the analysis of the central B-scans more difficult. Considering these limitations, it would be desirable if OCT systems for clinical use were further developed with a better probe head configuration and better light intensity to offset the attenuation effect with greater distances. Despite the imaging limitations, a 29 ± 72 µm mean change in enamel thickness, which was relatively small but significant, was detected over time, confirming the suitability of using CP-OCT to monitor ETW longitudinally. The detection of only a minimal change in enamel thickness was consistent with the relatively small change in BEWE scores.

Both BEWE and enamel thickness changed significantly over time, suggesting that both parameters could independently detect the clinical progression of ETW. However, in terms of correlation, neither enamel thickness nor change in enamel thickness correlated with BEWE or with a change in BEWE scores. There were also no significant differences in the changes in enamel thickness when comparing teeth that showed and did not show changes in BEWE (Table 4). This might be because a much larger area of the tooth was considered for the BEWE scoring while CP-OCT measured only a comparatively smaller area for enamel thickness measurements. Furthermore, the two parameters measured two different aspects of ETW severity on the enamel. BEWE scoring is performed depending on the surface area involved, whereas enamel thickness measurements reflect surface loss as a function of depth into the enamel relative to the original surface. These results suggest that it would be beneficial to assess ETW severity not just with BEWE but also with the enamel thickness assessment. Future studies should focus on which of the two measures of ETW severity correlates better with the onset of clinical symptoms resulting from ETW progression.

Among the 3D surface texture outcomes, only Sa (roughness) significantly correlated with BEWE_Buccal_ both at baseline and at M12 (Table 2). BEWE_Buccal_ scores were lower at baseline than at M12 (Table 1), which means ETW was less severe at baseline than the latter. Enamel surface roughness (Sa) was also lower at baseline compared to M12 (Table 1), corroborating the results of a previous in situ study that found higher surface roughness in more severe ETW lesions [13]. Several limitations of this exploratory clinical study, however, precluded further differentiation of the degree of surface roughness according to BEWE scores. This could be a focus of future investigations to see if certain thresholds for Sa values could correspond with ETW severity according to BEWE. Both dental microwear texture parameters Sa and Str (anisotropy) increased over time from baseline to M12. The increase in roughness was consistent with the previous study [13], while the Str trend was different as it did not previously change significantly. Asfc did not show any significant change in this study, contrary to our previous in situ study [13], where it significantly increased in the severe ETW group. ΔAsfc, ΔSa, and ΔStr all had a statistically significant but weak correlation with ΔBEWE_Buccal_ (Table 3), while none of the changes in all three surface texture parameters correlated with the change in enamel thickness. Teeth with an increase in BEWE had significantly greater ΔSa and ΔStr than teeth that did not show BEWE progression. This was expected as both BEWE and surface texture parameters measure changes on the enamel surface, in contrast to enamel thickness measurements, which relate more to cumulative surface loss. Of the three microwear texture parameters, only roughness (Sa) consistently increased with ETW progression over time in the current and previous in situ study [13]. These results might support the use of the surface roughness (Sa) parameter as another objective measure of ETW progression. Surface texture assessment was originally planned to be conducted at baseline, M3, M6, M9, and M12. However, due to the limitations mentioned earlier, the baseline and M12 measurements were prioritized, with the intermediate time points dropped from this study. 

## 5. Conclusions

Despite the several limitations of this exploratory study to objectively diagnose and monitor ETW clinically, the potential of surface roughness (Sa) as an objective outcome measure to identify ETW lesion severity and progression, comparable with the BEWE index, was confirmed. Moreover, longitudinal monitoring of enamel thickness using CP-OCT may be clinically relevant in addition to ETW severity determination by BEWE and or dental microwear parameters, accepting both tested hypotheses. Future studies resulting in a greater degree of ETW natural progression would be beneficial to further confirm our findings that surface roughness and enamel thickness are potentially suitable and appropriate objective measures for the clinical assessment and monitoring of erosive tooth wear.

## Figures and Tables

**Table 1:** Erosive tooth wear (ETW) outcomes (mean and standard deviation) at baseline and 12 months in dry-mouth subjects.

ETW Outcomes	Baseline	12 Months	*p*-Value
Asfc (complexity) ^a^	1.25 (0.80)	2.35 (5.94)	0.857
Sa (roughness) ^b^	207.04 (121.24)	375.61 (213.62)	<0.001
Str (anisotropy) ^c^	0.48 (0.17)	1.23 (1.35)	<0.001
Enamel Thickness ^d^	1056 (247)	1042 (249)	<0.001
BEWE_Buccal_	0.88 (0.61)	1.08 (0.48)	<0.001
BEWE_Occlusal_	0.82 (0.69)	1.38 (0.52)	<0.001
BEWE_Lingual_	0.64 (0.56)	1.13 (0.46)	<0.001
BEWE_Max_	0.99 (0.63)	1.33 (0.56)	<0.001

^a^ Asfc—area-scale fractal complexity (no unit); ^b^ Sa (nm); ^c^ Str—texture aspect ratio (no unit); ^d^ enamel thickness (µm).

**Table 2:** Overall correlations between ETW outcome measurements at baseline and M12.

ETW Outcomes		Baseline			M12		
		*n*	r	*p*-Value	*n*	r	*p*-Value
Asfc	Sa	584	0.63	<0.001	440	0.04	0.464
	Str	584	0.12	0.005	473	−0.42	<0.001
	Enamel Thickness	506	−0.02	0.676	491	−0.08	0.090
	BEWE_Buccal_	584	−0.11	0.006	506	0.09	0.046
Sa	Str	584	0.27	<0.001	391	0.22	<0.001
	Enamel Thickness	506	−0.01	0.889	417	0.09	0.071
	BEWE_Buccal_	584	−0.14	0.001	428	−0.13	0.007
Str	Enamel Thickness	506	0.07	0.104	445	0.17	<0.001
	BEWE_Buccal_	584	−0.04	0.398	459	−0.14	0.002
Enamel Thickness	BEWE_Buccal_	514	0.03	0.431	531	−0.01	0.859
	BEWE_Occlusal_	120	−0.19	0.040	129	−0.28	0.002
BEWE_Buccal_	BEWE_Occlusal_	150	0.18	0.025	135	0.05	0.533
	BEWE_Lingual_	568	0.42	<0.001	523	0.21	<0.001
	BEWE_Max_	597	0.86	<0.001	558	0.64	<0.001
BEWE_Occlusal_	BEWE_Lingual_	157	0.58	<0.001	145	*	*
	BEWE_Max_	157	0.50	<0.001	146	0.75	<0.001
BEWE_Lingual_	BEWE_Max_	595	0.55	<0.001	562	0.66	<0.001

* Correlation was not computed because all observations with both measurements have BEWE_Lingual_ = 1.

**Table 3:** Overall correlations between change (Δ) (M12–baseline) in ETW outcome measurements.

ETW Outcomes		*n*	r	*p*-Value
ΔAsfc	ΔSa	429	0.17	0.001
	ΔStr	460	−0.34	<0.001
	ΔEnamel Thickness	430	0.09	0.065
	ΔBEWE_Buccal_	494	−0.15	0.001
	ΔBEWE_Occlusal_	117	0.10	0.302
	ΔBEWE_Max_	507	−0.10	0.018
ΔSa	ΔStr	380	0.23	<0.001
	ΔEnamel Thickness	373	0.09	0.075
	ΔBEWE_Buccal_	418	0.16	0.001
	ΔBEWE_Occlusal_	100	−0.18	0.066
	ΔBEWE_Max_	428	0.03	0.531
ΔStr	ΔEnamel Thickness	387	0.02	0.643
	ΔBEWE_Buccal_	447	0.16	<0.001
	ΔBEWE_Occlusal_	103	−0.19	0.056
	ΔBEWE_Lingual_	430	−0.08	0.120
	ΔBEWE_Max_	458	−0.04	0.370
ΔEnamel Thickness	ΔBEWE_Buccal_	472	0.03	0.473
ΔBEWE_Buccal_	ΔBEWE_Occlusal_	129	0.15	0.100
	ΔBEWE_Lingual_	518	0.32	<0.001
	ΔBEWE_Max_	553	0.61	<0.001
ΔBEWE_Occlusal_	ΔBEWE_Lingual_	141	0.47	<0.001
	ΔBEWE_Max_	141	0.60	<0.001
ΔBEWE_Lingual_	ΔBEWE_Max_	559	0.61	<0.001

**Table 4:** Comparisons between teeth with vs without BEWE changes (M12–baseline).

ETW Outcomes	BEWE Increase	*n*	Mean (Standard Deviation)	*p*-Value
ΔAsfc (complexity) ^a^	No	362	0.60 (4.28)	0.770
	Yes	132	0.92 (6.97)	
ΔSa (roughness) ^b^	No	309	137.32 (251.12)	0.006
	Yes	109	234.42 (246.48)	
ΔStr (anisotropy) ^c^	No	322	0.65 (1.28)	<0.001
	Yes	125	1.28 (1.45)	
ΔEnamel Thickness ^d^	No	364	−26.44 (83.84)	0.725
	Yes	108	−23.33 (67.97)	

^a^ Asfc—area-scale fractal complexity (no unit); ^b^ Sa (nm); ^c^ Str—texture aspect ratio (no unit); ^d^ enamel thickness (µm).

## Data Availability

The data presented in this study are available upon request from the corresponding author.

## Supplementary Materials

The following supporting information can be downloaded at: https://www.mdpi.com/article/10.3390/diagnostics13152568/s1, Table S1: Erosive tooth wear (ETW) outcomes (mean and standard deviation) at baseline and 12 months in control subjects.
