# Supplementary material for: Exploratory Analysis of Objective Outcome Measures for the Clinical Assessment of Erosive Tooth Wear
Source: Diagnostics (Basel). 2023 Aug 2;13(15):2568. doi: 10.3390/diagnostics13152568 (PMC10417370; doi:10.3390/diagnostics13152568)
Supplement: Supplementary file 1 [file diagnostics-13-02568-s001.zip › diagnostics-2502769-supplementary.pdf]

SUPPLEMENTARY MATERIAL

Table S1. Erosive tooth wear (ETW) outcomes (mean and standard deviation) for baseline and 12 months in control subjects.

ETW Outcomes	Baseline	12 months
Asfc (complexity)[a]	0.94 (0.43)	0.64 (0.60)
Sa (roughness)[b]	124.43 (49.68)	360.23 (277.84)
Str (anisotropy)[c]	0.45 (0.15)	1.81 (1.13)
Enamel Thickness[d]	945 (202)	1040 (269)
$BEWE_{Buccal}$	0.92 (0.80)	1.19 (0.39)
$BEWE_{Occlusal}$	0.29 (0.61)	1.25 (0.45)
$BEWE_{Lingual}$	0.35 (0.53)	1.17 (0.38)
$BEWE_{Max}$	0.93 (0.80)	1.30 (0.46)

a: Asfc – area scale fractal complexity (no unit); b: Sa (nm); c: Str – texture aspect ratio (no unit); d: Enamel thickness (µm)
